# Hybrid nanofluid flow through a spinning Darcy–Forchheimer porous space with thermal radiation

**DOI:** 10.1038/s41598-021-95989-2

**Published:** 2021-08-18

**Authors:** Anwar Saeed, Muhammad Jawad, Wajdi Alghamdi, Saleem Nasir, Taza Gul, Poom Kumam

**Affiliations:** 1 Center of Excellence in Theoretical and Computational Science (TaCS-CoE), Faculty of Science, King Mongkut’s University of Technology Thonburi (KMUTT), 126 Pracha Uthit Rd., Bang Mod, Thung Khru, Bangkok, 10140 Thailand; 2 Department of Mathematics, University of Swabi, Swabi, 23430 Khyber Pakhtunkhwa Pakistan; 3 Department of Information Technology, Faculty of Computing and Information Technology, King Abdulaziz University, Jeddah, 80261 Saudi Arabia; 4 Department of Mathematics, City University of Science and Information Technology, Peshawar, 25000 Khyber Pakhtunkhwa Pakistan; 5 Department of Medical Research, China Medical University Hospital, China Medical University, Taichung, 40402 Taiwan

**Keywords:** Engineering, Mathematics and computing

## Abstract

This work investigates numerically the solution of Darcy–Forchheimer flow for hybrid nanofluid by employing the slip conditions. Basically, the fluid flow is produced by a swirling disk and is exposed to thermal stratification along with non-linear thermal radiation for controlling the heat transfer of the flow system. In this investigation, the nanoparticles of titanium dioxide and aluminum oxide have been suspended in water as base fluid. Moreover, the Darcy–Forchheimer expression is used to characterize the porous spaces with variable porosity and permeability. The resulting expressions of motion, energy and mass transfer in dimensionless form have been solved by HAM (Homotopy analysis method). In addition, the influence of different emerging factors upon flow system has been disputed both theoretically in graphical form and numerically in the tabular form. During this effort, it has been recognized that velocities profiles augment with growing values of mixed convection parameter while thermal characteristics enhance with augmenting values of radiation parameters. According to the findings, heat is transmitted more quickly in hybrid nanofluid than in traditional nanofluid. Furthermore, it is estimated that the velocities of fluid $f^{\prime}\left( \xi \right),g\left( \xi \right)$ are decayed for high values of $\phi_{1} ,\phi_{2} ,\,Fr$ and $k_{1}$ factors.

## Introduction

Nanofluids research has got immense consideration of academicians due to several technological and industrial uses. As a result of a variety of improved energy exchange uses, nanofluids are a serious fascinating and interesting issue of inspection in different sectors of engineering and technology. According to recent research, Nanofluids' heat transmission strength is far higher than that of conventional liquids. Thus, the replacement of ordinary fluids through nanoliquids is more secure. A few researchers and designers are pulled into the investigation of nanofluids on account of their greater energy capacities and utilizations. Nanofluids are renowned for providing substantially higher thermal conductivity than many other solvents. Nanofluids have had a significant impact on developing technologies and applications in the fields of research and innovation, medical science, and engineering. Researchers and scientists have utilized a variety of models to explore the thermal and mechanical properties of nanofluids. The idea of the nanomaterial addition with the classical fluid to augment its thermal conductivity was introduced for the first time by Choi^1^. The MHD flow has various uses in different industrial processes, like in nuclear energy reactors, crystal growth, electronic and electrical devices, solar energy technology, magnetic confinement fusion, and so on. Khan et al.^2^ described the expression of nanoliquid stream through a swinging sheet. In this direction, several scientists^3,4^ have studied nanofluid flow from several aspects. However, during the last few years, energy exchange in carbon nanoliquids has attracted a lot of attention from scientists in various disciplines. Carbon Nanotubes are a basic chemical formation with a carbon atom composition that is coiled into a cylindrical shape. It has been noted that the shape has always a strong influence on the thermal conduction of nanofluid. Also, Hatami et al.^5^ discussed the incompressible viscoelastic laminar motion of fluid owing to spinning and expanding discs. Mustafa et al.^6^ described the stream of liquid throughout the existence of nanoparticles by stretching the disk. They found that standardized disk stretching has a supreme role in reducing the thickness of the boundary layer. Pak and Cho^7^ studied experimentally the impacts of γ-alumina (Al_2_O_3_) and titanium dioxide on the turbulent heat energy transportation of water. These authors found that the mixing of nanoparticles with water fallouts in the enhancement of the convective heat energy transformation coefficient. Lunde et al.^8^ used Tiwari and Das model to perform the stability analysis and to discover the different solutions during the hybrid nanoliquid flow through a dwindling surface. Uddin et al.^9–13^ studied the radiative convectional flow of nanofluids using different configurations in presence of slip effect.

The hybrid nanoliquid is a special variant of nanofluid in which two or more different nanoscale materials are distributed in a working fluid in varying configurations. The nanomaterials configurations are selected with the goal of incorporating the beneficial effects including both nanomaterials into a single stable homogeneous system. The field of hybrid nanoliquids is growing rapidly. Hybrid nanoliquids have a wide spectrum of uses, which include modern automation cooling systems, automobile heat dissipation, hybrid electrical systems, fuel cells, gas sensing, bio-medicine manufacturing, renewable power, solar thermal, transistors, and domestic freezers. Researchers are interested in hybrid nanoliquids because of their growing demand in the heat transfer process. Few developments in hybrid nanofluid flow can be checked through Refs.^14,15^. Acharya et al.^16^ scrutinized a hybrid model to analyze the influence of hall current of two-dimensional flow over a revolving disk under the effect of thermal radiation. In this regard, a few of the significant and noteworthy study is highlighted in^17–20^.

For the last few decades, the theory of MHD is extremely appreciated for the various engineering and scientific purposes. It is actually the combination of fluid velocity with magnetic field. Such well-organized fact was first applied for different problems related to geophysics and astrophysics. In recent times, the MHD flow and heat exchange have achieved vital roles in agronomic engineering, industry of petroleum and medical field. In this setting, Davidson^21^, offered several applications appearing in the various fields of medical and engineering as well due to which MHD has grown the consideration of scientist. The exploration for flow and heat transfer of an electrical directed solution inside the scope of an attracting area through a heated surface has applications in accumulating events such as the excluding of polymerization, nuclear reactors, and the freezing of metal surface, and many others. The MHD two-layer electro-osmotic circulation involving entropy generation via micro-parallel units was explored by Xie and Jian^22^. Khan et al.^23^ analyzed the 3-dimensional steady MHD flow of Powell–Eyring nanofluids in convection and particles mass flux circumstances. Ellahi et al.^24^ explored energy exchange in a boundary layer flow having Magneto-hydrodynamic and entropy generation consequences. Ramzan et al.^25^ reported impressive findings of nanofluid flow over a stretchable medium in the presence of thermal radiation and MHD. They determined that as compared to inclined and vertical magnetic fields, the vertical magnetic field deflates the stream function significantly.

The boundary layer of mix convectional flows has many markets uses like dominant nuclear reactors, solar receivers, heat exchangers and electronic devices^26^. In light of these uses, a number of studies were conducted to determine the effects of mix convectional on nanofluid boundary layer flow. Hayat et al.^27^ used Cattaneo-Christov concept to quantitatively analyze the Darcy–Forchhemier flow on an expanding bending medium. Gul et al.^28^ considered the hybrid nanofluids (CNTs nanoparticles) flow through a swirling disk. Waini et al.^29^ employed hybrid nanofluids flowing on a vertical needle to investigate flow and heat.

Thermal radiation in the fluid flow further enhances the thermal efficiency of the hybrid nanofluid and is continuously used in the linear form^30,31^. In fact, in hybrid nanofluids, the volume fraction of the nanoparticles is limited up to 5%, and thermal radiations in linear form properly work. Even the researchers used the linear thermal radiation term in non-Newtonian fluids^32–35^ whose stress is not linear. While in the case of non-Newtonian fluids the nonlinear thermal radiation also exists and is very limited^36–38^. Haider et al.^38^ have used the Darcy–Forchheimer flow using the same model for various aspect of the physical parameters.

The ultimate priority of proposed study is to numerically analyze the solution of Darcy–Forchheimer flow for a hybrid nanofluid (${\text{Al}}_{2} {\text{O}}_{3} ,\;\,{\text{TiO}}_{2}$) by employing the slip condition. The flow is produced by a swirling disk and is exposed to thermal stratification and nonlinear thermal radiation for controlling the heat transfer of the flow system. In this investigation, the nanoparticles of titanium dioxide and aluminum oxide have been suspended in water as base fluid. Moreover, the Darcy–Forchheimer expression is used to characterize the porous spaces with variable porosity and permeability. The published work^38^ is also extended with the addition of concentration profile. The Brownian motion and Thermophoresis analysis have also been used to extend the existing literature^38^. The resulting expressions have been solved by HAM (homotopy analysis method). The significance of different emerging factors upon flow system has been discussed both theoretically in graphical form and numerically in the tabular form.

## Problem formulation

**Figure 1:**
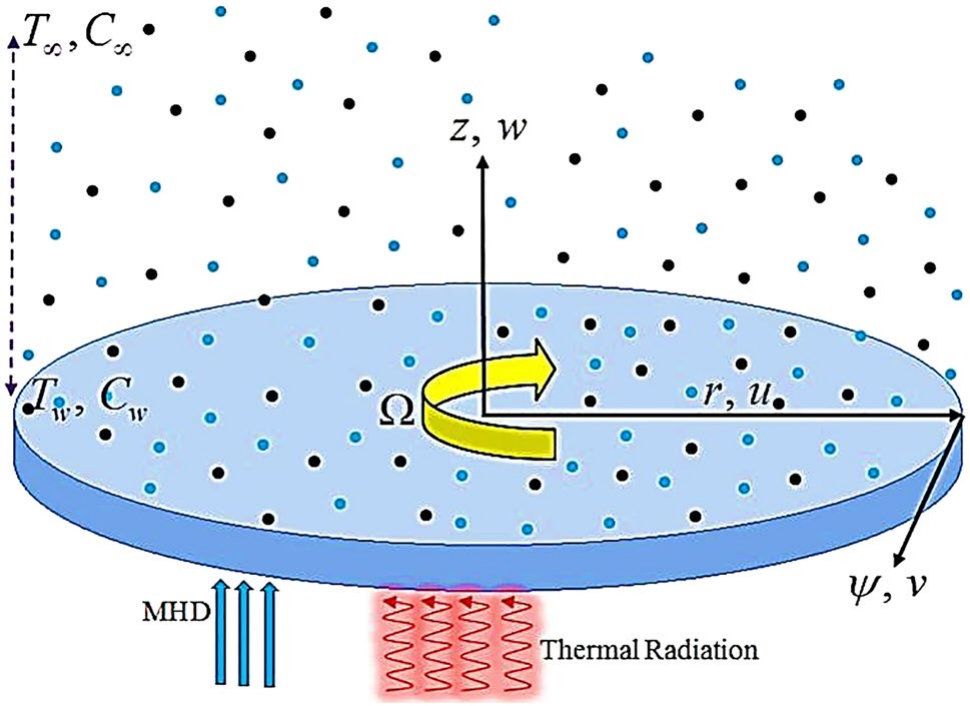
The schematic layout of 3-dimensional problem.

We assume that the time independent three-dimensional (3D) flow of hybrid nanofluid with velocity slip by a rotating disk, as depicted in Fig. 1. Thermal stratification, heat generation/absorption and non-linear thermal radiation are also accounted. The constant angular velocity of turning disk is $\Omega$. The components of velocity in the ascending orientations are $\left( {r,\psi ,z} \right)$ & $\left( {u,v,w} \right)$. Using Buongiorno model, the three-dimensional flow resulting equations in the aforementioned conditions is given as^27,38^:

1 $$ \frac{\partial \,u}{{\partial \,r}} + \,\frac{u}{r}\,\, + \frac{\partial \,w}{{\partial \,z}} = 0, $$

2 $$ u\left( {\frac{\partial u}{{\partial r}}} \right) - \frac{{v^{2} }}{r} + w\left( {\frac{\partial u}{{\partial z}}} \right) = \upsilon_{hnf} \left( {\frac{{\partial^{2} u}}{{\partial r^{2} }} + \frac{1}{r}\,\,\frac{\partial u}{{\partial r}} - \frac{u}{{r^{2} }} + \frac{{\partial^{2} u}}{{\partial z^{2} }}} \right) - \frac{{\upsilon_{hnf} \varepsilon \left( z \right)}}{K\left( z \right)}u - \frac{{C_{b} \varepsilon^{2} \left( z \right)}}{{\sqrt {K\left( z \right)} }}u\sqrt {u^{2} + v^{2} } , $$

3 $$ u\left( {\frac{\partial v}{{\partial r}}} \right) + \frac{uv}{r} + w\left( {\frac{\partial u}{{\partial z}}} \right) = \upsilon_{hnf} \left( {\frac{{\partial^{2} v}}{{\partial r^{2} }} - \frac{v}{{r^{2} }} + \frac{1}{r}.\frac{\partial v}{{\partial r}} + \frac{{\partial^{2} v}}{{\partial z^{2} }}} \right) - \frac{{\upsilon_{hnf} \varepsilon \left( z \right)}}{K\left( z \right)}v - \frac{{C_{b} \varepsilon^{2} \left( z \right)}}{{\sqrt {K\left( z \right)} }}v\sqrt {u^{2} + v^{2} } , $$

4 $$ u\left( {\frac{\partial w}{{\partial r}}} \right) + w\left( {\frac{\partial w}{{\partial z}}} \right) = \upsilon_{hnf} \left( {\frac{{\partial^{2} w}}{{\partial r^{2} }} + \frac{1}{r}\frac{\partial w}{{\partial r}} + \frac{{\partial^{2} w}}{{\partial z^{2} }}} \right) - \frac{{\upsilon_{hnf} \varepsilon \left( z \right)}}{K\left( z \right)}w - \frac{{C_{b} \varepsilon^{2} \left( z \right)}}{{\sqrt {K\left( z \right)} }}w\sqrt {u^{2} + v^{2} } , $$

5 $$ \begin{aligned} u\frac{\partial T}{{\partial r}} + w\frac{\partial T}{{\partial z}} & = \alpha_{hnf} \left( {\frac{{\partial^{2} T}}{{\partial r^{2} }} + \frac{1}{r}\frac{\partial T}{{\partial r}} + \frac{{\partial^{2} T}}{{\partial z^{2} }}} \right) - \frac{1}{{\left( {\rho cp} \right)_{hnf} }}\frac{{\partial q_{r} }}{\partial z}  \\ & \quad + \tau_{hnf} \left[ {D_{B} \left( {\frac{\partial C}{{\partial z}}\frac{\partial T}{{\partial z}}} \right) + \frac{{D_{T} }}{{{\rm T}_{\infty } }}\left( {\frac{\partial T}{{\partial z}}} \right)^{2} } \right] \hfill + \frac{Q}{{\left( {\rho c_{p} } \right)_{hnf} }}\left( {T - T_{\infty } } \right), \hfill \\ \end{aligned} $$

6 $$ \left[ {u\frac{\partial C}{{\partial r}} + w\frac{\partial C}{{\partial z}}} \right] = D_{B} \frac{{\partial^{2} C}}{{\partial z^{2} }} + \left( {\frac{{D_{T} }}{{{\rm T}_{\infty } }}} \right)\frac{{\partial^{2} {\rm T}}}{{\partial z^{2} }}, $$

7 $$ \begin{gathered} u = L\left( {\frac{\partial u}{{\partial z}}} \right),\,\,v = r\,\Omega + L\left( {\frac{\partial v}{{\partial z}}} \right),\,\,w = 0,\,\,T = T_{w} \, = T_{0} + Ar,\,\,C = C_{w} \,\,at\,z = 0, \hfill \\ u \to 0,\,\,\,\,v \to 0,\,\,\,T \to T_{\infty } = T_{0} + Br,\,\,\,C \to C_{\infty } ,\,\,\,\,at\,z = \infty . \hfill \\ \end{gathered} $$

where form^27,30^

8 $$ K\left( z \right) = K_{\infty } \left( {1 + de^{\frac{z}{r}} } \right), $$

9 $$ \varepsilon \left( z \right) = \varepsilon_{\infty } \left( {1 + d^{*} e^{\frac{z}{r}} } \right), $$

10 $$ q_{r} = - \frac{{4\sigma^{*} }}{3k}\frac{{\partial T^{4} }}{\partial z} = - \frac{{16\sigma^{*} }}{3k}T^{3} \left( {\frac{\partial T}{{\partial z}}} \right), $$

Here $C_{b}$ (Drag coefficient), $d^{*}$ (variable porosity), $T_{0}$ (Reference temperature), $\sigma^{*}$ (Stefan Boltzmann constant), $k$ (coefficient of Mean absorption), $d$ (Variable permeability), $T_{\infty }$ (at free stream Thermal stratification), $L_{1}$ (Velocity slip coefficient), $T_{w}$ (At wall Thermal stratification), $A$ and $B$ dimensional constants, $K_{\infty }$ (permeability) and $\varepsilon_{\infty }$ (porosity). Thus, the energy equation develops

11 $$ \begin{aligned} u\frac{\partial T}{{\partial r}} + w\frac{\partial T}{{\partial z}} & = \alpha_{hnf} \left( {\frac{{\partial^{2} T}}{{\partial r^{2} }} + \frac{1}{r}\frac{\partial T}{{\partial r}} + \frac{{\partial^{2} T}}{{\partial z^{2} }}} \right) + \frac{1}{{\left( {\rho cp} \right)_{hnf} }}\frac{{16\sigma^{*} }}{3k}\frac{\partial }{\partial z}\left( {T^{3} \frac{\partial T}{{\partial z}}} \right) \hfill \\ & \quad + \tau_{hnf} \left[ {D_{B} \left( {\frac{\partial C}{{\partial z}}\frac{\partial T}{{\partial z}}} \right) + \frac{{D_{T} }}{{{\rm T}_{\infty } }}\left( {\frac{\partial T}{{\partial z}}} \right)^{2} } \right] + \frac{Q}{{\left( {\rho c_{p} } \right)_{hnf} }}\left( {T - T_{\infty } } \right), \hfill \\ \end{aligned} $$

Theoretic model for hybrid nanofluid is^14^**:**

12 $$ \begin{aligned} \mu_{hnf} & = \frac{{\mu_{f} }}{{\left( {1 - \phi_{1} - \phi_{2} } \right)^{2.5} }},\nu_{hnf} = \frac{{\mu_{hnf} }}{{\rho_{hnf} }},\rho_{hnf} = \left( {1 - \phi_{1} - \phi_{2} } \right)\left( \rho \right)_{f} + \phi_{1} \left( \rho \right)_{1} + \phi_{2} \left( \rho \right)_{2} , \hfill \\ \alpha_{hnf} & = \frac{{k_{hnf} }}{{\left( {\rho c_{p} } \right)_{hnf} }},\left( {\rho c_{p} } \right)_{hnf} = \left( {1 - \phi_{1} - \phi_{2} } \right)\,\,\left( {\rho \,c_{p} } \right)_{f} + \phi_{1} \,\left( {\rho \,c_{p} } \right)_{1} + \phi_{2} \left( {\rho \,c_{p} } \right)_{2} , \hfill \\ \frac{{k_{nf} }}{{k_{f} }} & = \frac{{k_{1} \,\phi_{1} + k_{2} \,\phi_{2} + 2\phi \,k_{f} + 2\phi \,\,\left( {\phi_{1} k_{1} + \phi_{2} k_{2} } \right) - 2\,\,\left( {\phi_{1} + \phi_{2} } \right)^{2} \,\,k_{f} }}{{k_{1} \,\phi_{1} + k_{2} \,\phi_{2} + 2\phi \,k_{f} + \phi \,\,\left( {\phi_{1} \,k_{1} + \phi_{2} \,k_{2} } \right) + \,\,\left( {\phi_{1} + \phi_{2} } \right)^{2} \,\,k_{f} }}. \hfill \\ \end{aligned} $$

In the preceding formulations, the subscript *hnf* and *f* denoted the hybrid nanofluid and base fluid. $k_{hnf}$ (Thermal conductivity), $\phi_{1}$ (Volume fraction of ${\text{TiO}}_{2}$), $k_{2}$ (Thermal conductivity of ${\text{Al}}_{2} {\text{O}}_{3}$), $\phi_{2}$ (Solid volume fraction of ${\text{Al}}_{2} {\text{O}}_{3}$), $\mu_{hnf}$ (Effective dynamic viscosity), $\left( {\rho c_{p} } \right)_{hnf}$ (Heat capacity), $k_{f}$ (Thermal conductivity), $\left( \rho \right)_{hnf}$ (Density), $k_{1}$ the thermal conductivity of ${\text{TiO}}_{2}$, $\rho_{1}$ the density of ${\text{TiO}}_{2}$, $\rho_{2}$ the density of ${\text{Al}}_{2} {\text{O}}_{3}$, and $\rho_{f}$ the density of base fluid. Thermo physical features of nanoparticles and water are showed in Table 1^15^ below.

**Table 1:** Thermo physical features of nanoparticles and water^15^.

Physical properties	Nanoparticles		Base fluid
	${\text{TiO}}_{2}$	${\text{Al}}_{2} {\text{O}}_{3}$	
$k\left( {{\text{W}}/{\text{mk}}} \right)$	8.4	0.613	0.613
$c_{p} \left( {{\text{J}}/{\text{kg}}\;{\text{K}}} \right)$	692	4179	4179
$\rho \left( {{\text{kg}}/{\text{m}}^{3} } \right)$	4230	997.1	997.1

Considering

13 $$ \begin{aligned} u & = r\Omega f^{\prime}\left( \xi \right),v = \Omega g\left( \xi \right),w = - \sqrt {2\Omega v_{f} } f\left( \xi \right), \hfill \\ \Theta \left( \xi \right) &  = \frac{{T - T_{\infty } }}{{T_{w} - T_{\infty } }},\Phi \left( \xi \right) = \frac{{C - C_{\infty } }}{{C_{w} - C_{\infty } }},\xi = \left( {\frac{2\Omega }{{v_{f} }}} \right)^{1/2} z, \hfill \\ \end{aligned} $$

with $T = \left( {T_{w} - T_{0} } \right)\theta \left( \xi \right) + T_{\infty }$ Eq. (1) is identically proved and Eqs. (2)−(11) yield

14 $$ \begin{gathered} \frac{1}{{\left( {1 - \phi_{1} - \phi_{2} } \right)\left( {1 - \phi_{1} - \phi_{2} + \frac{{\rho_{1} }}{{\rho_{f} }}\phi_{1} + \frac{{\rho_{2} }}{{\rho_{f} }}\phi_{2} } \right)}}\left( {2f^{\prime\prime\prime} - \frac{1}{{2k_{1} {\text{Re}}_{r} }}\left( {\frac{{1 + d^{*} e^{ - \xi } }}{{1 + de^{ - \xi } }}} \right)f^{\prime}} \right) \hfill \\ - F_{r} \left( {\frac{{1 + d^{*} e^{ - \xi } }}{{\sqrt {1 + de^{ - \xi } } }}} \right)\left( {f^{{\prime}{2}} + \frac{1}{2}g^{2} } \right) - f^{{\prime}{2}} + g^{2} + 2ff^{\prime\prime} = 0, \hfill \\ \end{gathered} $$

15 $$ \begin{gathered} \frac{1}{{\left( {1 - \phi_{1} - \phi_{2} } \right)\left( {1 - \phi_{1} - \phi_{2} + \frac{{\rho_{1} }}{{\rho_{f} }}\phi_{1} + \frac{{\rho_{2} }}{{\rho_{f} }}\phi_{2} } \right)}}\left( {2g^{\prime\prime} - \frac{1}{{2k_{1} {\text{Re}}_{r} }}\left( {\frac{{1 + d^{*} e^{ - \xi } }}{{1 + de^{ - \xi } }}} \right)g} \right) \hfill \\ - F_{r} \left( {\frac{{1 + d^{*} e^{ - \xi } }}{{\sqrt {1 + de^{ - \xi } } }}} \right)\left( {g^{2} + \frac{1}{2}f^{{\prime}{2}} } \right) - f^{\prime}g + fg^{\prime} = 0, \hfill \\ \end{gathered} $$

16 $$ \begin{gathered} \frac{1}{{\left( {1 - \phi_{1} - \phi_{2} } \right)\left( {1 - \phi_{1} - \phi_{2} + \frac{{\left( {\rho cp} \right)_{1} }}{{\left( {\rho cp} \right)_{f} }}\phi_{1} + \frac{{\left( {\rho cp} \right)_{2} }}{{\left( {\rho cp} \right)_{f} }}\phi_{2} } \right)}}\left[ {\left( {\frac{{k_{hnf} }}{{k_{f} }} + \frac{4}{3}R\left[ {\left( {\frac{1}{{\Theta_{w} + s_{t} }}} \right)\Theta + 1} \right]^{3} } \right)\Theta^{\prime}} \right]^{\prime } + \Pr f\Theta^{\prime} \hfill \\ + \frac{{\frac{{\left( {\rho cp} \right)_{hnp} }}{{\left( {\rho cp} \right)_{p} }}}}{{\frac{{\left( {\rho cp} \right)_{hnf} }}{{\left( {\rho cp} \right)_{f} }}}}\left[ {N_{b} \Theta^{\prime}\phi^{\prime} + N_{t} \Theta^{{\prime}{2}} } \right] + \frac{{k_{f} }}{{k_{hnf} }}\alpha \Theta^{\prime} = 0, \hfill \\ \end{gathered} $$

17 $$ \Phi^{\prime\prime} - \Pr Scf\Phi^{\prime} + \frac{{N_{t} }}{{N_{b} }}\Theta^{\prime\prime} = 0, $$

18 $$ \begin{gathered} f = 0,\,\,f^{\prime} = \gamma \,f^{\prime\prime},g = 1 + \gamma \,g^{\prime},\,\,\Theta = 1 - S_{t} ,\Phi = 1\,\,\,\,at\,\,\xi = 0 \hfill \\ f^{\prime} \to 0,\,\,\,g \to 0,\,\,\Theta \to 0\,,\Phi \to 0,\,\,\,at\,\,\,\,\xi \to \infty . \hfill \\ \end{gathered} $$

Here $\left( {k_{1} } \right)$ the porosity factor, $\left( {Pe_{r} } \right)$ the Peclet number, $Nt$ thermophoresis parameter, $Nb$ Brownian motion parameter, $\left( {{\text{Re}}_{r} } \right)$ the local Reynolds number, $\left( {F_{r} } \right)$ the local inertial factor, $\left( R \right)$ radiation factor, $\left( {\Pr } \right)$ Prandtl number, $\left( {S_{t} } \right)$ the thermal stratification factor, Schmidt number $\left( {Sc} \right),$ and heat source factor $\left( \alpha \right)$ defined by:

19 $$ \begin{gathered} \gamma = L\left( {\frac{2\Omega }{{\upsilon_{f} }}} \right)^{1/2} ,k_{1} = \frac{{K_{\infty } }}{{r^{2} \varepsilon_{\infty } }},{\text{Re}}_{r} = \frac{{U_{w} r}}{\upsilon },F_{r} = \frac{{C_{b} \varepsilon_{\infty }^{2} }}{\sqrt K }r,Sc = \frac{\upsilon }{{D_{B} }},Nt = \frac{{D_{T} (T_{w} - T_{\infty } )}}{{\upsilon T_{\infty } }}, \hfill \\ Nb = \frac{{\tau D_{B} (C_{w} - C_{\infty } )}}{\upsilon },S_{t} = \frac{B}{A},R = \frac{{4\sigma^{*} T_{\infty }^{3} }}{{kk_{f} }},\Pr = \frac{{v_{f} }}{{\alpha_{f} }},Pe_{r} = {\text{Re}}_{r} \Pr ,\alpha = \frac{Q}{{\Omega \left( {\rho c_{p} } \right)_{f} }}. \hfill \\ \end{gathered} $$

### Quantities of physical interest

Significant physical factors such as $C_{f} ,\,C_{g} ,\,Nu$ and $Sh$ are expressed for engineering purposes as

20 $$ \left. \begin{aligned} \left[ {Re_{r} } \right]^{\frac{1}{2}} C_{f} & = \frac{1}{{\left( {1 - \phi_{1} - \phi_{2} } \right)^{2.5} }}f^{\prime\prime}\left( 0 \right), \hfill \\ \left[ {Re_{r} } \right]^{\frac{1}{2}} C_{g} & = \frac{1}{{\left( {1 - \phi_{1} - \phi_{2} } \right)^{2.5} }}g^{\prime}\left( 0 \right) \hfill \\ \frac{1}{2}\left[ {Re_{r} } \right]^{{ - \frac{1}{2}}} Nu & = - \left( {\frac{{k_{hnf} }}{{k_{f} }} + \frac{4}{3}R\left( {\frac{1}{{\theta_{w} + S_{t} }} + 1} \right)^{3} } \right)\theta^{\prime}\left( 0 \right), \hfill \\ \frac{1}{2}\left[ {Re_{r} } \right]^{{ - \frac{1}{2}}} Sh & = - \Phi \left( 0 \right) \hfill \\ \end{aligned} \right\} $$

## Solution by homotopy analysis method

Equations (13–16) with specified boundary conditions Eq. (17) are tackled through the HAM^39–43^. Mathematica software is used for this goal.

21 $$ L_{{\overset{\lower0.5em\hbox{$\smash{\scriptscriptstyle\frown}$}}{f} }} \,(\overset{\lower0.5em\hbox{$\smash{\scriptscriptstyle\frown}$}}{f} ) = \overset{\lower0.5em\hbox{$\smash{\scriptscriptstyle\frown}$}}{f}^{\prime\prime\prime},\,\,\,\,L_{{\overset{\lower0.5em\hbox{$\smash{\scriptscriptstyle\frown}$}}{g} }} \,(\overset{\lower0.5em\hbox{$\smash{\scriptscriptstyle\frown}$}}{g} ) = \overset{\lower0.5em\hbox{$\smash{\scriptscriptstyle\frown}$}}{g}^{\prime\prime},\,\,\,\,\,{\text{L}}_{{\overset{\lower0.5em\hbox{$\smash{\scriptscriptstyle\frown}$}}{\theta } }} {(}\overset{\lower0.5em\hbox{$\smash{\scriptscriptstyle\frown}$}}{\Theta } {) = }\overset{\lower0.5em\hbox{$\smash{\scriptscriptstyle\frown}$}}{\Theta }^{\prime\prime} \, ,\,\,\,\,\,{\text{L}}_{{\overset{\lower0.5em\hbox{$\smash{\scriptscriptstyle\frown}$}}{\Phi } }} {(}\overset{\lower0.5em\hbox{$\smash{\scriptscriptstyle\frown}$}}{\Phi } {) = }\overset{\lower0.5em\hbox{$\smash{\scriptscriptstyle\frown}$}}{\Phi^{\prime\prime}} , $$

The linear operators are defined as:

22 $$ \begin{gathered} L_{{\overset{\lower0.5em\hbox{$\smash{\scriptscriptstyle\frown}$}}{f} }} \,\,(e_{1} + e_{2} \eta + e_{3} \eta^{2} ) = 0,\,\,\,\,{\text{L}}_{{\overset{\lower0.5em\hbox{$\smash{\scriptscriptstyle\frown}$}}{g} }} \,\,(e_{6} + e_{7} \eta ) = 0, \hfill \\ {\text{L}}_{{\overset{\lower0.5em\hbox{$\smash{\scriptscriptstyle\frown}$}}{\theta } }} \,\,(e_{8} + e_{9} \eta ) = 0 \, ,\,\,\,\,\,\,{\text{L}}_{{\overset{\lower0.5em\hbox{$\smash{\scriptscriptstyle\frown}$}}{\Phi } }} \,\,(e_{10} + e_{11} \eta ) = 0. \hfill \\ \end{gathered} $$

The non-linear operatives are chosen as $${\rm N}_{{\overset{\lower0.5em\hbox{$\smash{\scriptscriptstyle\frown}$}}{f} }} ,{\rm N}_{{\overset{\lower0.5em\hbox{$\smash{\scriptscriptstyle\frown}$}}{g} }} ,{\rm N}_{{\overset{\lower0.5em\hbox{$\smash{\scriptscriptstyle\frown}$}}{\Theta } }} \,and\,\,{\rm N}_{{\overset{\lower0.5em\hbox{$\smash{\scriptscriptstyle\frown}$}}{\Phi } }} \,$$ and identify in system:

23 $$ \begin{aligned} \, {\rm N}_{{\overset{\lower0.5em\hbox{$\smash{\scriptscriptstyle\frown}$}}{f} }} \, \left[ {\overset{\lower0.5em\hbox{$\smash{\scriptscriptstyle\frown}$}}{f} (\xi ;\,\,\zeta ),\overset{\lower0.5em\hbox{$\smash{\scriptscriptstyle\frown}$}}{g} (\xi ;\,\,\zeta )} \right] & = \,\,\frac{1}{{\left( {1 - \phi_{1} - \phi_{2} } \right)\left( {1 - \phi_{1} - \phi_{2} + \frac{{\rho_{1} }}{{\rho_{f} }}\phi_{1} + \frac{{\rho_{2} }}{{\rho_{f} }}\phi_{2} } \right)}} \hfill \\&\quad \left( {2\overset{\lower0.5em\hbox{$\smash{\scriptscriptstyle\frown}$}}{f}_{\xi \xi \xi } - \frac{1}{{2k_{1} {\text{Re}}_{r} }}\left( {\frac{{1 + d^{*} e^{ - \xi } }}{{1 + de^{ - \xi } }}} \right)\overset{\lower0.5em\hbox{$\smash{\scriptscriptstyle\frown}$}}{f}_{\xi } } \right) - F_{r} \left( {\frac{{1 + d^{*} e^{ - \xi } }}{{\sqrt {1 + de^{ - \xi } } }}} \right)\left( {\overset{\lower0.5em\hbox{$\smash{\scriptscriptstyle\frown}$}}{f}_{\xi }^{2} + \frac{1}{2}\overset{\lower0.5em\hbox{$\smash{\scriptscriptstyle\frown}$}}{g}^{2} } \right) - \overset{\lower0.5em\hbox{$\smash{\scriptscriptstyle\frown}$}}{f}_{\xi }^{2} \hfill \\&\quad + \overset{\lower0.5em\hbox{$\smash{\scriptscriptstyle\frown}$}}{g}^{2} + 2\overset{\lower0.5em\hbox{$\smash{\scriptscriptstyle\frown}$}}{f} \overset{\lower0.5em\hbox{$\smash{\scriptscriptstyle\frown}$}}{f}_{\xi \xi \xi } , \hfill \\ \,\,\,\,\,\,\,\,\,\,\, \hfill \\ \end{aligned} $$

24 $$ \begin{aligned} {\rm N}_{{\overset{\lower0.5em\hbox{$\smash{\scriptscriptstyle\frown}$}}{g} }} \, \left[ {\overset{\lower0.5em\hbox{$\smash{\scriptscriptstyle\frown}$}}{f} (\xi ;\,\,\zeta ),\overset{\lower0.5em\hbox{$\smash{\scriptscriptstyle\frown}$}}{g} (\xi ;\,\,\zeta )} \right] & = \,\frac{1}{{\left( {1 - \phi_{1} - \phi_{2} } \right)\left( {1 - \phi_{1} - \phi_{2} + \frac{{\rho_{1} }}{{\rho_{f} }}\phi_{1} + \frac{{\rho_{2} }}{{\rho_{f} }}\phi_{2} } \right)}} \hfill \\&\quad \left( {2\overset{\lower0.5em\hbox{$\smash{\scriptscriptstyle\frown}$}}{g}_{\xi \xi } - \frac{1}{{2k_{1} {\text{Re}}_{r} }}\left( {\frac{{1 + d^{*} e^{ - \xi } }}{{1 + de^{ - \xi } }}} \right)\overset{\lower0.5em\hbox{$\smash{\scriptscriptstyle\frown}$}}{g} } \right) - F_{r} \left( {\frac{{1 + d^{*} e^{ - \xi } }}{{\sqrt {1 + de^{ - \xi } } }}} \right)\left( {\overset{\lower0.5em\hbox{$\smash{\scriptscriptstyle\frown}$}}{g}^{2} + \frac{1}{2}\overset{\lower0.5em\hbox{$\smash{\scriptscriptstyle\frown}$}}{f}_{\xi }^{2} } \right) - \overset{\lower0.5em\hbox{$\smash{\scriptscriptstyle\frown}$}}{f}_{\xi } \overset{\lower0.5em\hbox{$\smash{\scriptscriptstyle\frown}$}}{g} + \overset{\lower0.5em\hbox{$\smash{\scriptscriptstyle\frown}$}}{f} \overset{\lower0.5em\hbox{$\smash{\scriptscriptstyle\frown}$}}{g}_{\xi } , \hfill \\ \end{aligned} $$

25 $$ \begin{gathered} {\rm N}_{{\overset{\lower0.5em\hbox{$\smash{\scriptscriptstyle\frown}$}}{\Theta } }} \left[ {\overset{\lower0.5em\hbox{$\smash{\scriptscriptstyle\frown}$}}{\Theta } (\xi ;\zeta ),\overset{\lower0.5em\hbox{$\smash{\scriptscriptstyle\frown}$}}{f} (\xi ;\zeta )} \right] = \frac{1}{{\left( {1 - \phi_{1} - \phi_{2} } \right)\left( {1 - \phi_{1} - \phi_{2} + \frac{{\left( {\rho_{cp} } \right)_{1} }}{{\left( {\rho_{cp} } \right)_{f} }}\phi_{1} + \frac{{\left( {\rho_{cp} } \right)_{2} }}{{\left( {\rho_{cp} } \right)_{f} }}\phi_{2} } \right)}} \hfill \\ \left[ {\left( {\frac{{k_{hnf} }}{{k_{f} }} + \frac{4}{3}R\left[ {\left( {\frac{1}{{\theta_{w} + s_{t} }}} \right)\overset{\lower0.5em\hbox{$\smash{\scriptscriptstyle\frown}$}}{\Theta } + 1} \right]^{3} } \right)\overset{\lower0.5em\hbox{$\smash{\scriptscriptstyle\frown}$}}{\theta }_{\xi } } \right]_{\xi } + \Pr \overset{\lower0.5em\hbox{$\smash{\scriptscriptstyle\frown}$}}{f} \overset{\lower0.5em\hbox{$\smash{\scriptscriptstyle\frown}$}}{\theta }_{\xi } + \frac{{k_{f} }}{{k_{hnf} }}\alpha \overset{\lower0.5em\hbox{$\smash{\scriptscriptstyle\frown}$}}{\Theta } , \hfill \\ \end{gathered} $$

26 $$ {\rm N}_{{\overset{\lower0.5em\hbox{$\smash{\scriptscriptstyle\frown}$}}{\Phi } }} \left[ {\overset{\lower0.5em\hbox{$\smash{\scriptscriptstyle\frown}$}}{\Theta } (\xi ;\zeta ),\overset{\lower0.5em\hbox{$\smash{\scriptscriptstyle\frown}$}}{\Phi } (\xi ;\zeta )} \right] = \overset{\lower0.5em\hbox{$\smash{\scriptscriptstyle\frown}$}}{\Phi }_{\xi \xi } - \Pr Sc\overset{\lower0.5em\hbox{$\smash{\scriptscriptstyle\frown}$}}{f} \overset{\lower0.5em\hbox{$\smash{\scriptscriptstyle\frown}$}}{\Phi }_{\xi } + \frac{{N_{t} }}{{N_{b} }}\overset{\lower0.5em\hbox{$\smash{\scriptscriptstyle\frown}$}}{\Theta }_{\xi \xi } , $$

While BCs are:

27 $$ \begin{gathered} \left. {\frac{{\partial \overset{\lower0.5em\hbox{$\smash{\scriptscriptstyle\frown}$}}{f} (\xi ;\zeta )}}{\partial \xi }} \right|_{\xi = 0} = \gamma \left. {\frac{{\partial^{2} \overset{\lower0.5em\hbox{$\smash{\scriptscriptstyle\frown}$}}{f} (\xi ;\zeta )}}{{\partial \xi^{2} }}} \right|_{\xi = 0} , \, \left. {\overset{\lower0.5em\hbox{$\smash{\scriptscriptstyle\frown}$}}{f} (\xi ;\zeta )} \right|_{\xi = 0} = 0,\left. {\overset{\lower0.5em\hbox{$\smash{\scriptscriptstyle\frown}$}}{g} (\xi ;\zeta )} \right|_{\eta = 0} = 1 + \gamma \left. {\frac{{\partial \overset{\lower0.5em\hbox{$\smash{\scriptscriptstyle\frown}$}}{g} (\xi ;\zeta )}}{\partial \eta }} \right|_{\xi = 0} , \hfill \\ \left. {\overset{\lower0.5em\hbox{$\smash{\scriptscriptstyle\frown}$}}{\Theta } (\xi ;\zeta )} \right|_{\xi = 0} = 1 - S_{t} ,\left. {\overset{\lower0.5em\hbox{$\smash{\scriptscriptstyle\frown}$}}{\Phi } (\xi ;\zeta )} \right|_{\xi = 0} = 1, \hfill \\ \left. {\frac{{\partial \overset{\lower0.5em\hbox{$\smash{\scriptscriptstyle\frown}$}}{f} (\eta ;\zeta )}}{\partial \xi }} \right|_{\xi = \infty } = 0,\left. {\overset{\lower0.5em\hbox{$\smash{\scriptscriptstyle\frown}$}}{g} (\xi ;\zeta )} \right|_{\xi = \infty } = 1,\left. {\overset{\lower0.5em\hbox{$\smash{\scriptscriptstyle\frown}$}}{\Theta } (\xi ;\zeta )} \right|_{\xi = \infty } = 0,\left. {\overset{\lower0.5em\hbox{$\smash{\scriptscriptstyle\frown}$}}{\Phi } (\xi ;\zeta )} \right|_{\xi = \infty } = 0. \hfill \\ \end{gathered} $$

Here,$$\zeta$$ is the embedding parameter $$\zeta \in [0,1]$$, to ensure that the convergence of the solution is consistent $$\hbar_{{\overset{\lower0.5em\hbox{$\smash{\scriptscriptstyle\frown}$}}{f} }} ,\hbar_{{\overset{\lower0.5em\hbox{$\smash{\scriptscriptstyle\frown}$}}{g} }}$$ and $$\hbar_{{\overset{\lower0.5em\hbox{$\smash{\scriptscriptstyle\frown}$}}{\theta } }}$$ is used. By choosing $$\zeta = 0{\text{ and }}\zeta = 1$$, we have

28 $$ \begin{gathered} \overset{\lower0.5em\hbox{$\smash{\scriptscriptstyle\frown}$}}{f} \left( {\xi ;1} \right) = \overset{\lower0.5em\hbox{$\smash{\scriptscriptstyle\frown}$}}{f} (\xi ), \hfill \\ \overset{\lower0.5em\hbox{$\smash{\scriptscriptstyle\frown}$}}{g} \left( {\xi ;1} \right) = \overset{\lower0.5em\hbox{$\smash{\scriptscriptstyle\frown}$}}{g} (\xi )\,,\,\,\, \hfill \\ \overset{\lower0.5em\hbox{$\smash{\scriptscriptstyle\frown}$}}{\Theta } (\xi ;1) = \overset{\lower0.5em\hbox{$\smash{\scriptscriptstyle\frown}$}}{\Theta } (\xi ) \, , \hfill \\ \overset{\lower0.5em\hbox{$\smash{\scriptscriptstyle\frown}$}}{\Phi } (\xi ;1) = \overset{\lower0.5em\hbox{$\smash{\scriptscriptstyle\frown}$}}{\Phi } (\xi ), \hfill \\ \end{gathered} $$

Develop the Taylor’s series for $$\overset{\lower0.5em\hbox{$\smash{\scriptscriptstyle\frown}$}}{f} (\xi ;\zeta ),\,\,\,\overset{\lower0.5em\hbox{$\smash{\scriptscriptstyle\frown}$}}{g} (\xi ;\zeta ),\,\,\,\overset{\lower0.5em\hbox{$\smash{\scriptscriptstyle\frown}$}}{\theta } (\xi ;\zeta )$$ and $$\overset{\lower0.5em\hbox{$\smash{\scriptscriptstyle\frown}$}}{\Phi } (\xi ;\zeta )$$ about the point $$\zeta = 0$$

29 $$ \begin{gathered} \overset{\lower0.5em\hbox{$\smash{\scriptscriptstyle\frown}$}}{f} (\xi ;\zeta ) = \overset{\lower0.5em\hbox{$\smash{\scriptscriptstyle\frown}$}}{f}_{0} (\xi ) + \sum\limits_{n = 1}^{\infty } {\,\,\overset{\lower0.5em\hbox{$\smash{\scriptscriptstyle\frown}$}}{f}_{n} (\xi )\zeta^{n} } , \hfill \\ \overset{\lower0.5em\hbox{$\smash{\scriptscriptstyle\frown}$}}{g} (\xi ;\zeta ) = \overset{\lower0.5em\hbox{$\smash{\scriptscriptstyle\frown}$}}{g}_{0} (\xi ) + \sum\limits_{n = 1}^{\infty } {\,\,\overset{\lower0.5em\hbox{$\smash{\scriptscriptstyle\frown}$}}{g}_{n} (\xi )\zeta^{n} } , \hfill \\ \overset{\lower0.5em\hbox{$\smash{\scriptscriptstyle\frown}$}}{\Theta } (\xi ;\zeta ) = \overset{\lower0.5em\hbox{$\smash{\scriptscriptstyle\frown}$}}{\Theta }_{0} (\xi ) + \sum\limits_{n = 1}^{\infty } {\,\,\overset{\lower0.5em\hbox{$\smash{\scriptscriptstyle\frown}$}}{\Theta }_{n} (\xi )\zeta^{n} } , \hfill \\ \overset{\lower0.5em\hbox{$\smash{\scriptscriptstyle\frown}$}}{\Phi } (\xi ;\zeta ) = \overset{\lower0.5em\hbox{$\smash{\scriptscriptstyle\frown}$}}{\Phi }_{0} (\xi ) + \sum\limits_{n = 1}^{\infty } {\,\,\overset{\lower0.5em\hbox{$\smash{\scriptscriptstyle\frown}$}}{\Phi }_{n} (\xi )\zeta^{n} } . \hfill \\ \end{gathered} $$

30 $$ \begin{gathered} \overset{\lower0.5em\hbox{$\smash{\scriptscriptstyle\frown}$}}{f}_{n} (\xi ) \, = \left. {\frac{1}{n!}.\frac{{\partial \overset{\lower0.5em\hbox{$\smash{\scriptscriptstyle\frown}$}}{f} \left( {\xi ;\zeta } \right)}}{\partial \xi }} \right|_{p = 0} ,\overset{\lower0.5em\hbox{$\smash{\scriptscriptstyle\frown}$}}{g}_{n} (\xi ) \, = \left. {\frac{1}{n!}.\frac{{\partial \overset{\lower0.5em\hbox{$\smash{\scriptscriptstyle\frown}$}}{g} \,\,\left( {\xi ;\,\,\,\zeta } \right)}}{\partial \,\xi }} \right|_{p = 0} ,\,\,\,\,\,\,\,\,\,\overset{\lower0.5em\hbox{$\smash{\scriptscriptstyle\frown}$}}{\Theta }_{n} (\xi ) \, = \left. {\frac{1}{n!}.\frac{{\partial \overset{\lower0.5em\hbox{$\smash{\scriptscriptstyle\frown}$}}{\Theta } \left( {\xi ;\zeta } \right)}}{\partial \xi }} \right|_{p = 0} \hfill \\ ,\,\,\overset{\lower0.5em\hbox{$\smash{\scriptscriptstyle\frown}$}}{\Phi }_{n} (\xi ) \, = \left. {\frac{1}{n!}.\frac{{\partial \overset{\lower0.5em\hbox{$\smash{\scriptscriptstyle\frown}$}}{\Phi } \left( {\xi ;\zeta } \right)}}{\partial \xi }} \right|_{p = 0} . \hfill \\ \, \hfill \\ \end{gathered} $$

While B.Cs are:

31 $$ \begin{gathered} \overset{\lower0.5em\hbox{$\smash{\scriptscriptstyle\frown}$}}{f} \left( 0 \right) = 0,\,\,\overset{\lower0.5em\hbox{$\smash{\scriptscriptstyle\frown}$}}{f^{\prime}} \left( 0 \right) = \gamma \,\overset{\lower0.5em\hbox{$\smash{\scriptscriptstyle\frown}$}}{f^{\prime\prime}} ,\,\,\overset{\lower0.5em\hbox{$\smash{\scriptscriptstyle\frown}$}}{g} \left( 0 \right) = 1 + \gamma \,\overset{\lower0.5em\hbox{$\smash{\scriptscriptstyle\frown}$}}{g^{\prime}} \left( 0 \right),\,\,\,\overset{\lower0.5em\hbox{$\smash{\scriptscriptstyle\frown}$}}{\Theta } \left( 0 \right) = 1 - S_{t} ,\,\,\,\overset{\lower0.5em\hbox{$\smash{\scriptscriptstyle\frown}$}}{\Phi } \left( 0 \right) = 1, \hfill \\ \overset{\lower0.5em\hbox{$\smash{\scriptscriptstyle\frown}$}}{f^{\prime}} \left( \infty \right) \to 0,\,\,\,\overset{\lower0.5em\hbox{$\smash{\scriptscriptstyle\frown}$}}{g} \left( \infty \right) \to 1,\,\,\,\overset{\lower0.5em\hbox{$\smash{\scriptscriptstyle\frown}$}}{\Theta } \left( \infty \right) \to 0,\,\,\,\overset{\lower0.5em\hbox{$\smash{\scriptscriptstyle\frown}$}}{\Phi } \left( \infty \right) \to 0. \hfill \\ \end{gathered} $$

## Results and discussion

**Figure 2:**
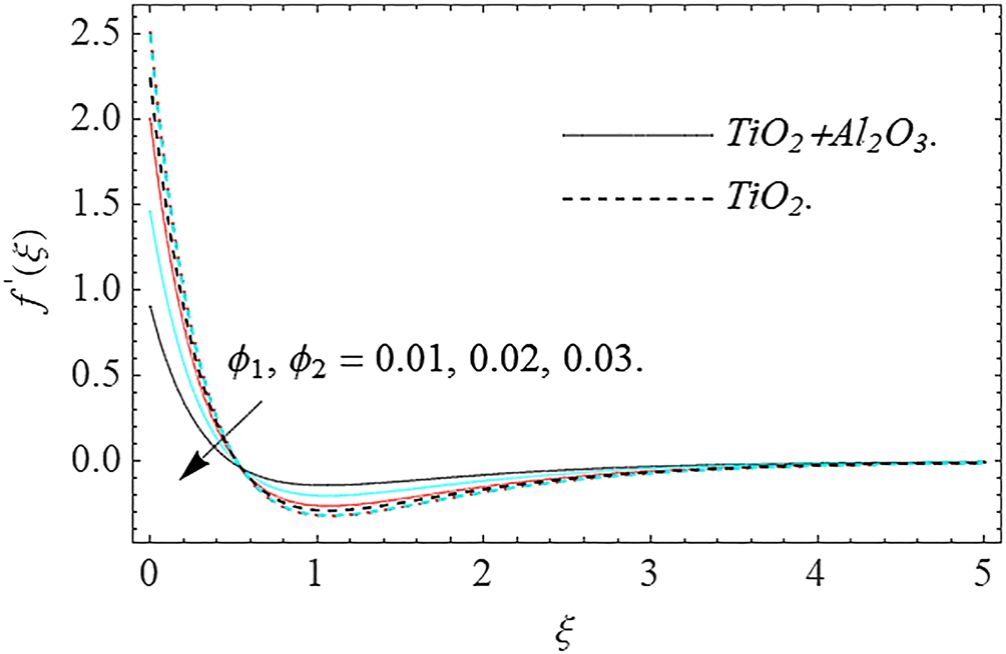
Velocity profile $f^{\prime}\left( \xi \right)\,$ against $\phi_{1} ,\phi_{2}$.

**Figure 3:**
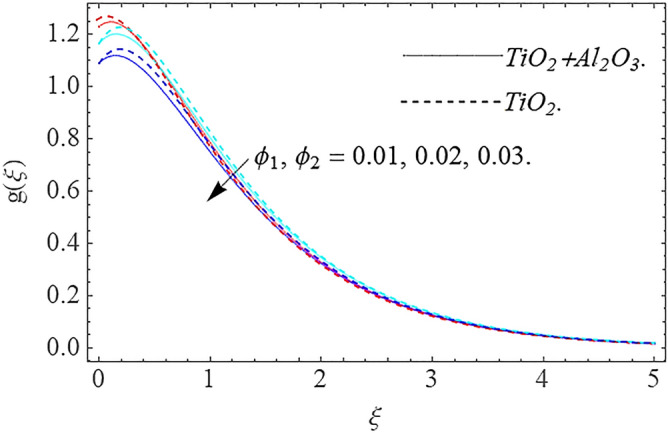
Velocity profile $g\left( \xi \right)\,$ against $\phi_{1} ,\phi_{2}$.

**Figure 4:**
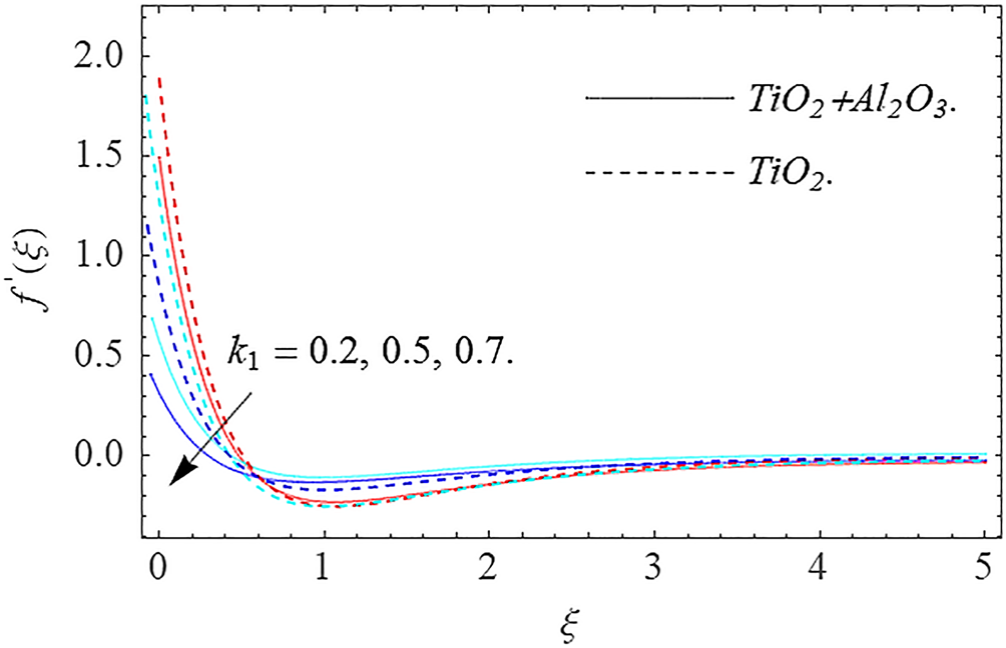
Velocity profile $f^{\prime}\left( \xi \right)\,$ against $k_{1}$.

**Figure 5:**
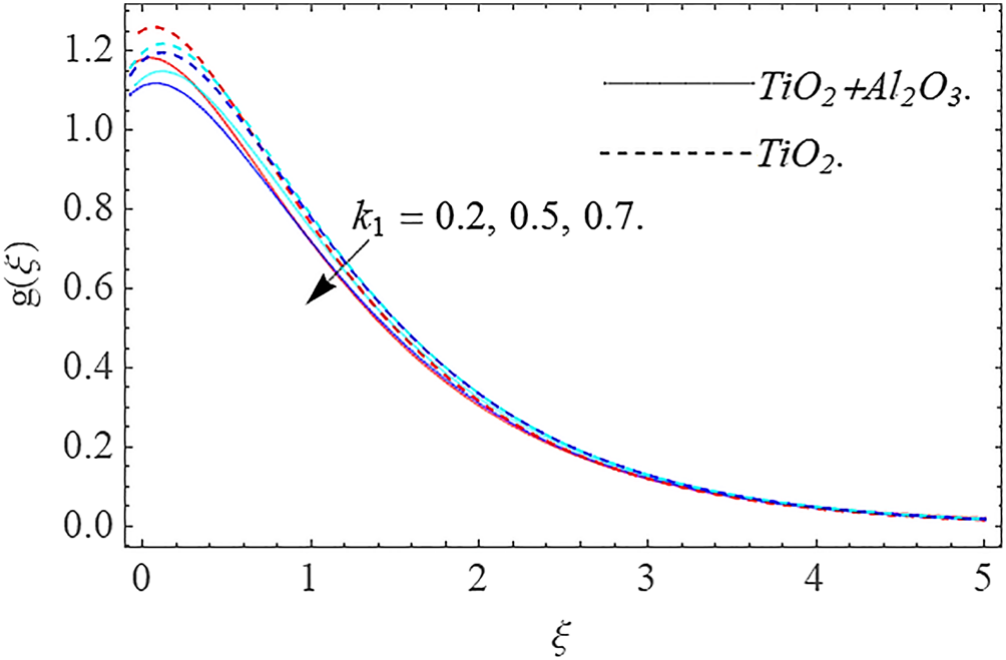
Velocity profile $g\left( \xi \right)\,$ against $k_{1}$.

**Figure 6:**
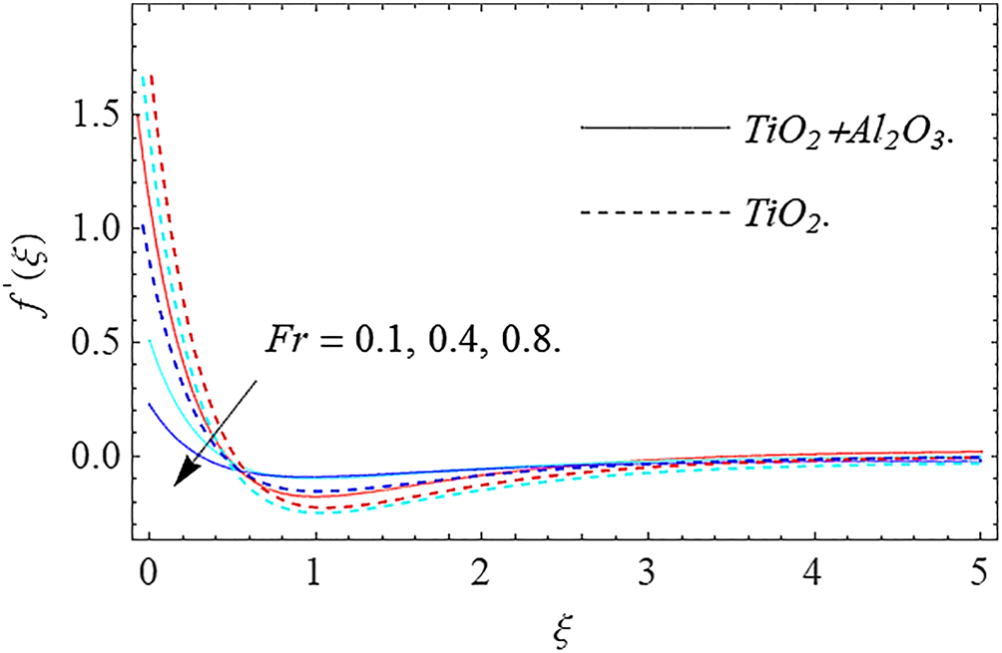
Velocity profile $f^{\prime}\left( \xi \right)\,$ against $Fr$.

**Figure 7:**
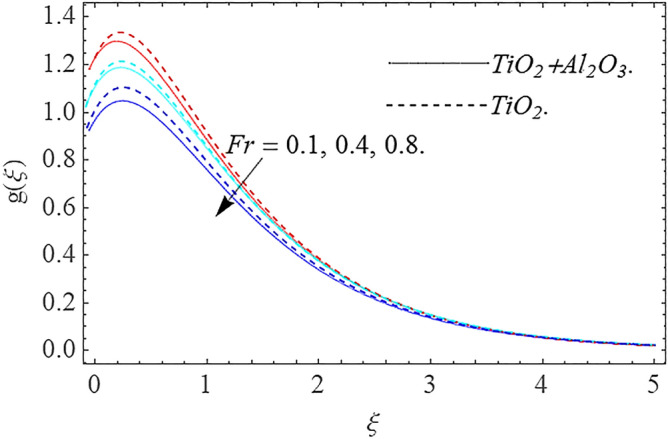
Velocity profile $g\left( \xi \right)\,$ against $Fr$.

**Figure 8:**
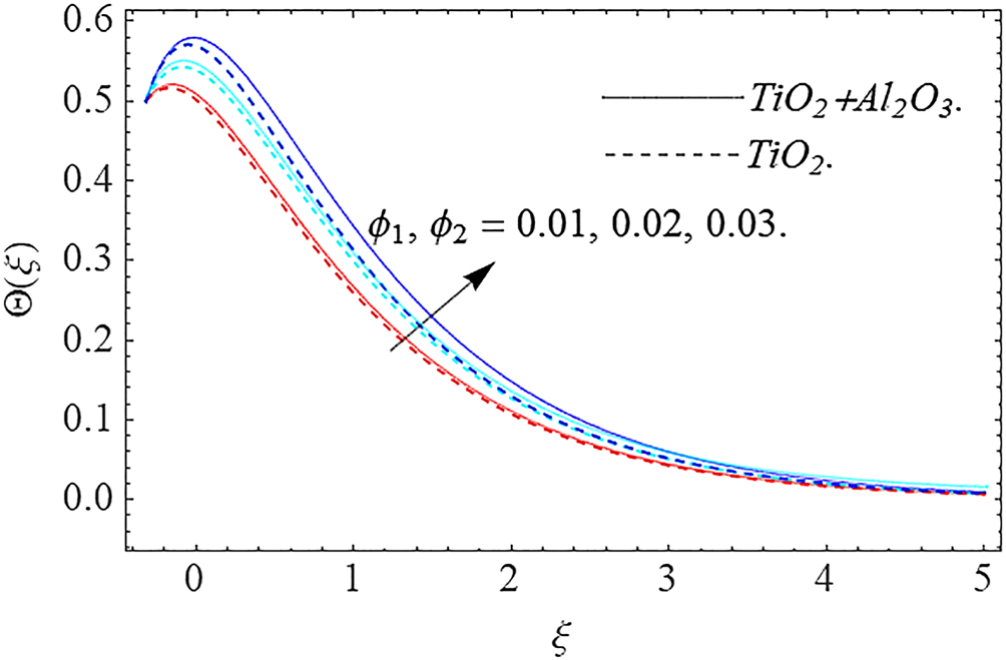
Thermal profile $\Theta (\xi )$ against $\phi_{1} ,\phi_{2}$.

**Figure 9:**
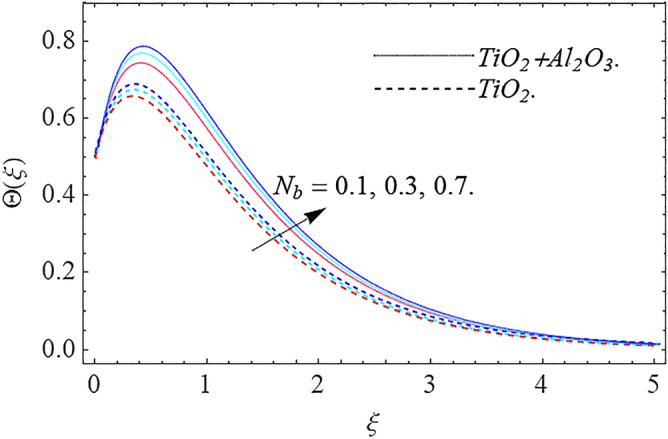
Thermal profile $\Theta (\xi )$ against $N_{b}$.

**Figure 10:**
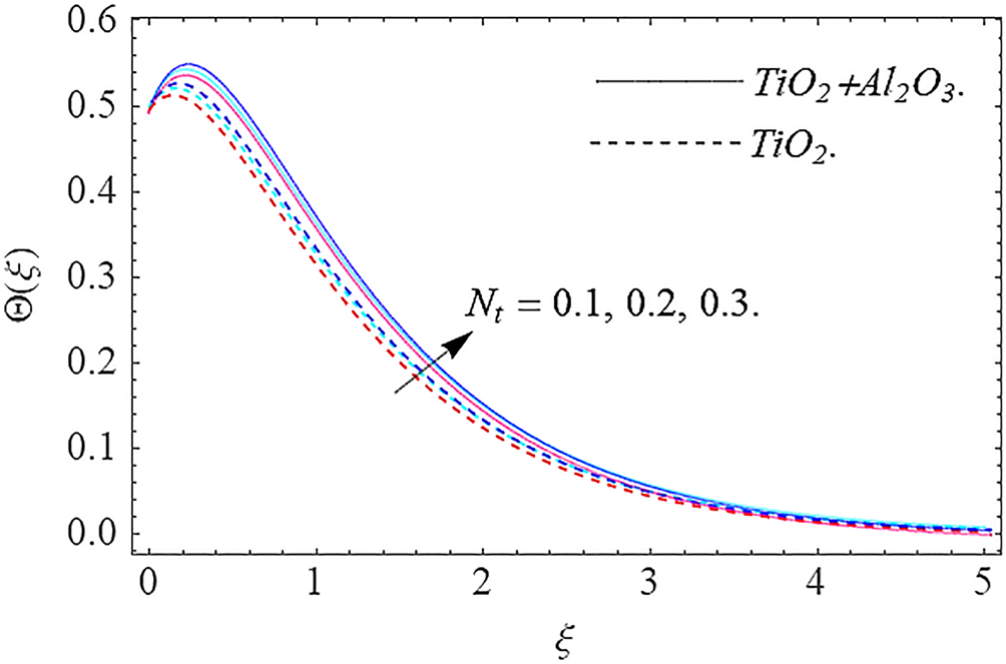
Thermal profile $\Theta (\xi )$ against $N_{t}$.

**Figure 11:**
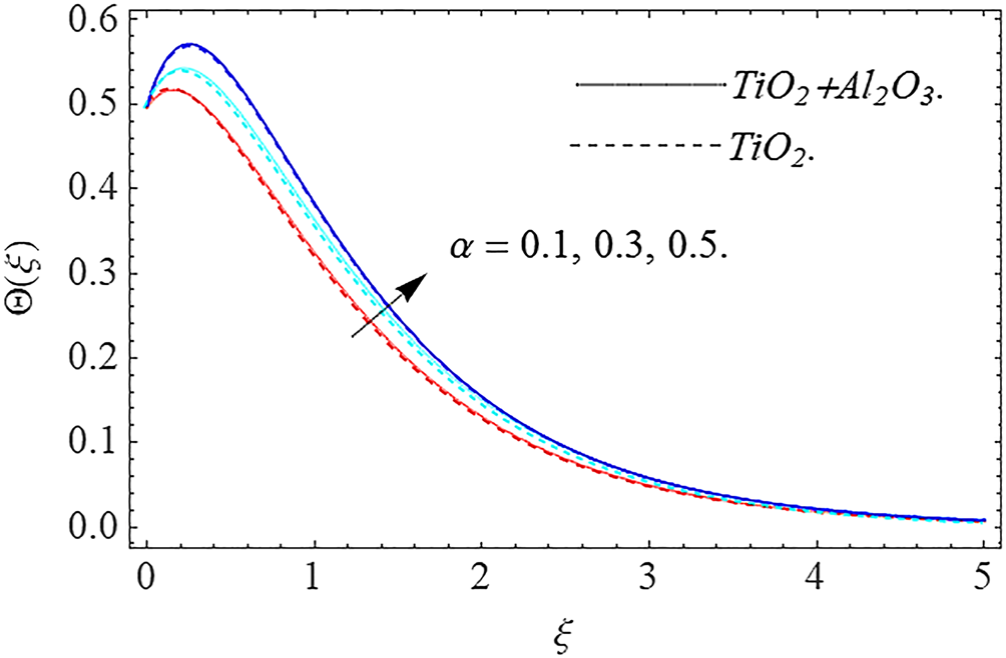
Thermal profile $\Theta (\xi )$ against $\alpha$.

**Figure 12:**
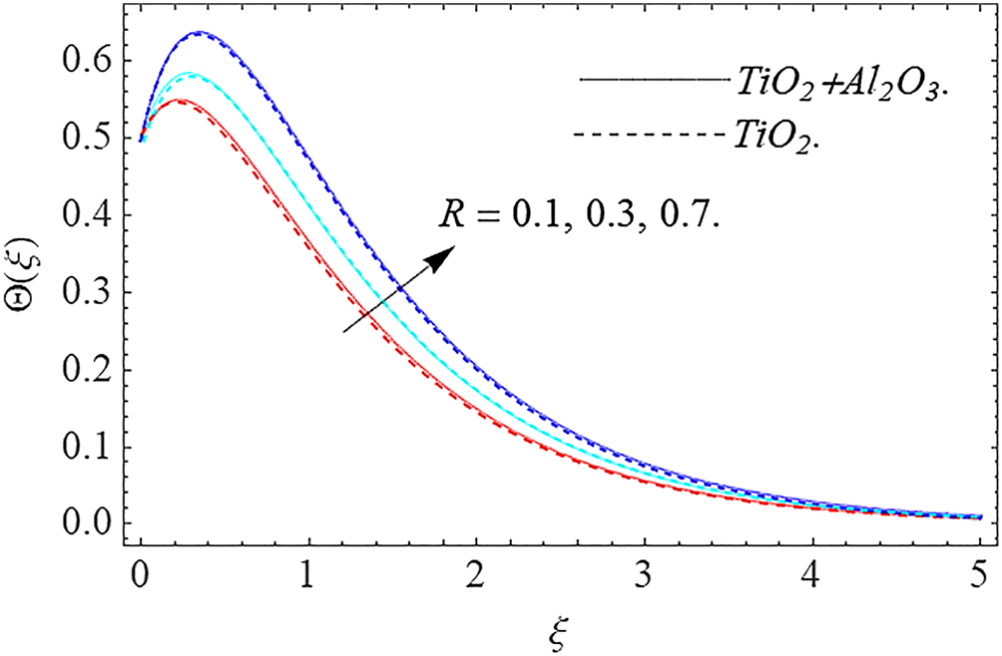
Thermal profile $\Theta (\xi )$ against $R$.

**Figure 13:**
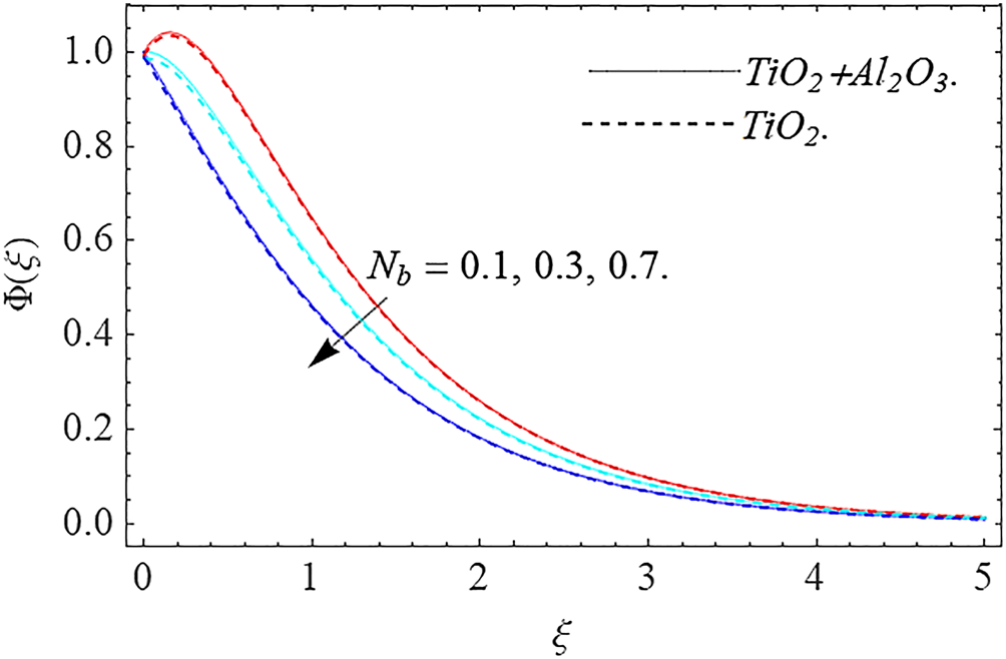
Concentration profile $\Phi (\xi )$ against $N_{b}$.

**Figure 14:**
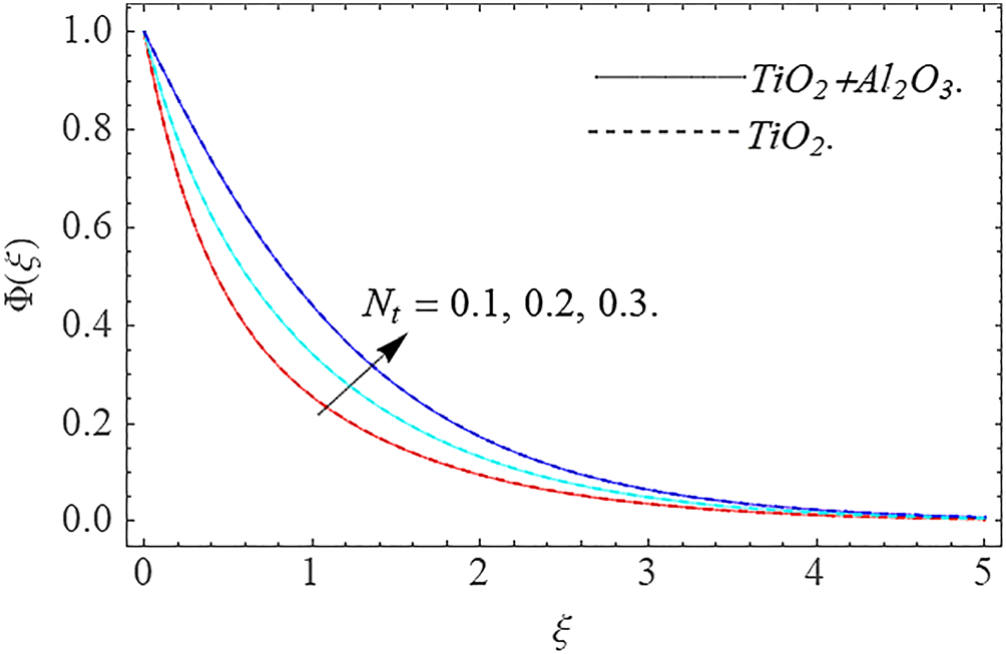
Concentration profile $\Phi (\xi )$ against $N_{t}$.

**Figure 15:**
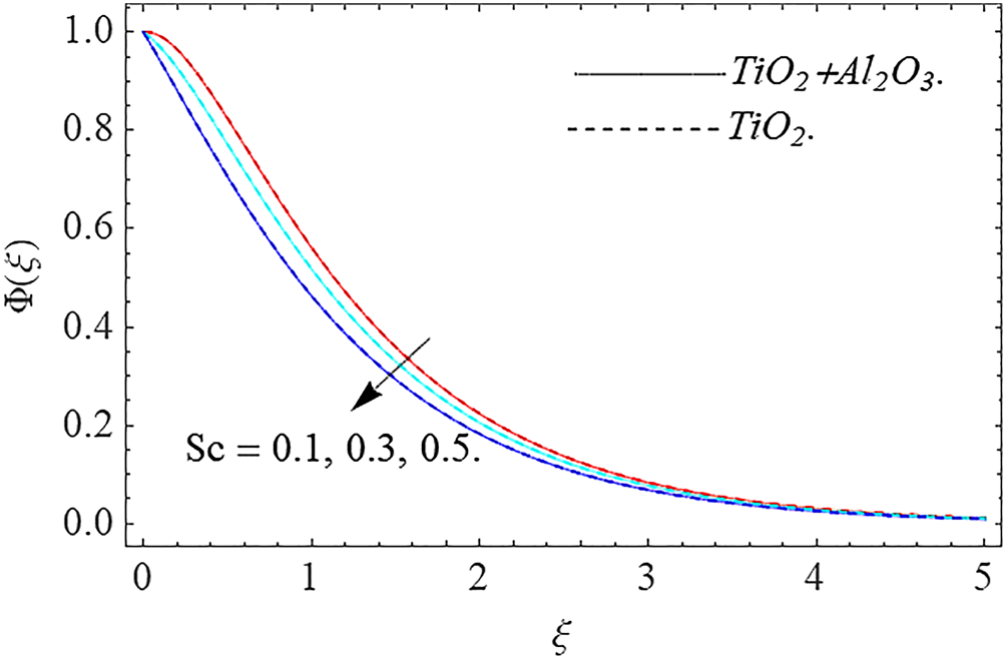
Concentration profile $\Phi (\xi )$ against $Sc$.

In this part, we address about the behavior of diverse emerging flow parameters such as $$\phi_{1} ,\phi_{2}$$, $${\text{Re}}_{r}$$, $$F_{r}$$, $$k_{1}$$, $$M$$, $$\theta_{w}$$, $$R$$, $$\left( {S_{t} } \right)$$ the thermal stratification parameter, and heat source parameter $$\left( \alpha \right)$$**.** The geometry of the model problem is shown in Fig. 1. Figures 2, 3, 4, 5, 6 and 7 highlight that how different values of relevant factors affect $${\text{TiO}}_{2}$$ nanofluid and $${\text{Al}}_{2} {\text{O}}_{3} + {\text{TiO}}_{2}$$ hybrid nanofluid $$f^{\prime}\left( \xi \right),g\left( \xi \right)$$ (velocity profiles). Figures 2 and 3 show the steady-state primary $$f^{\prime}\left( \xi \right)$$ and $$g\left( \xi \right)$$ secondary velocity distributions of $${\text{TiO}}_{2}$$ nanofluid and $${\text{Al}}_{2} {\text{O}}_{3} + {\text{TiO}}_{2}$$ hybrid nanofluid for varying $$\phi_{1} ,\phi_{2}$$ values. The graph indicated that the greater magnitudes of $$\phi_{1} ,\phi_{2}$$ causes the primary $$f^{\prime}\left( \xi \right)$$ and $$g\left( \xi \right)$$ secondary velocity distributions to drop. Physically, the collision of inter-particles intensifies as the $$\phi_{1} ,\phi_{2}$$ of $${\text{Al}}_{2} {\text{O}}_{3} ,\,\,{\text{TiO}}_{2}$$ improves, and as a response, the $$TiO_{2}$$ nanofluid and $${\text{Al}}_{2} {\text{O}}_{3} + {\text{TiO}}_{2}$$ hybrid nanofluid primary $$f^{\prime}\left( \xi \right)$$ and $$g\left( \xi \right)$$ secondary velocity distributions decreases. The primary $$f^{\prime}\left( \xi \right)$$ and $$g\left( \xi \right)$$ secondary velocity fluctuations for $$k_{1}$$ (porous media factor) for $${\text{TiO}}_{2}$$ nanofluid and $${\text{Al}}_{2} {\text{O}}_{3} + {\text{TiO}}_{2}$$ hybrid nanofluid are emphasized in Figs. 4 and 5. We can see from the graph that as the amplitude of $$k_{1}$$ increases, the primary $$f^{\prime}\left( \xi \right)$$ and $$g\left( \xi \right)$$ secondary velocity distributions diminish. Physically, increasing the size of $$k_{1}$$ causes a reduction in the transparency of the porous zone. As a response, there is a small dump in the surface of disk which oppose nanofluid and hybrid nanofluid to flow through, and the fluid velocity become slow. The fluctuations $$Fr$$ versus primary $$f^{\prime}\left( \xi \right)$$ and $$g\left( \xi \right)$$ secondary velocity distributions for both $${\text{TiO}}_{2}$$ nanofluid and $${\text{Al}}_{2} {\text{O}}_{3} + {\text{TiO}}_{2}$$ hybrid nanofluid are highlighted in Figs. 6 and 7. It should be emphasized from the plots that for nanofluid and hybrid nanofluid, $$Fr$$ is the decreasing function of both $$f^{\prime}\left( \xi \right)$$ and $$g\left( \xi \right)$$. In essence, a rise in $$Fr$$ causes fluids to become more resilient, leading to decreased $$f^{\prime}\left( \xi \right)$$ and $$g\left( \xi \right)$$. Physically, boosting the amplitude of F reduces the interior nanofluid velocity, but it has no effect on the liquid thicknesses. As a consequence, a rise in F creates a well stream resistance, limiting fluid velocity $$f^{\prime}\left( \xi \right)$$ and $$g\left( \xi \right)$$ distributions. Figures 8,9,10,11, and 12 illustrate how significant variation of key variables influence on $${\text{TiO}}_{2}$$ nanofluid and $${\text{Al}}_{2} {\text{O}}_{3} + {\text{TiO}}_{2}$$ hybrid nanofluid $$\theta \left( \xi \right)$$ thermal behavior. The steady-state $$\theta \left( \xi \right)$$ temperature distributions of $${\text{TiO}}_{2}$$ nanofluid and $${\text{Al}}_{2} {\text{O}}_{3} + {\text{TiO}}_{2}$$ hybrid nanofluid for various values of $$\phi_{1} ,\phi_{2}$$ are shown in Fig. 8. The plot observed that adding $$\phi_{1} 
,\phi_{2}$$ optimizes the thermal distributions. Physically, this trend is attributable to $${\text{TiO}}_{2}$$ and $${\text{Al}}_{2} {\text{O}}_{3} + {\text{TiO}}_{2}$$ greater thermal conductivity as $$\phi_{1} ,\phi_{2}$$ increases, that becomes the major source of temperature increase. Figure 9 exhibits the temperature curve for multiple $$N_{b}$$ values. The elevation in $$N_{b}$$ leads to a rise in $$\theta \left( \xi \right)$$ temperature distributions, as seen in Fig. 9. Such phenomena are associated with a significant increase in Brownian motion, that reveals the erratic motion of molecules dispersed in $${\text{TiO}}_{2}$$ nanofluid and $${\text{Al}}_{2} {\text{O}}_{3} + {\text{TiO}}_{2}$$ hybrid nanofluid. It may be deduced as enhancing the Brownian motion, boosts the temperature significantly across the boundary layer by raising the collision of $${\text{TiO}}_{2}$$ nanofluid and $${\text{Al}}_{2} {\text{O}}_{3} + {\text{TiO}}_{2}$$ hybrid nanofluid particles. The influence of multiple variations of the thermophoretic parameter $$N_{t}$$ on $${\text{TiO}}_{2}$$ nanofluid and $${\text{Al}}_{2} {\text{O}}_{3} + {\text{TiO}}_{2}$$ hybrid nanofluid $$\theta \left( \xi \right)$$ temperature profiles is depicted in Fig. 10. That's obvious to see how raising $$N_{t}$$ values improve the $$\theta \left( \xi \right)$$ across the boundary. In terms of physics, the increase in $$N_{t}$$ is due to an increase in the thermophoretic process. Thermophoresis is a form of molecular mobility that occurs when thermal gradients are imposed, and it is deeply linked to the soret phenomenon. Due to particle dispersion mediated by the thermophoretic phenomenon, nanomaterials transmit thermal energy from the heated edge to the coldest edge inside the boundary layer area. As a result, the temperature of the fluid increases rapidly. In the influence of buoyancy force, Fig. 11 depicts the $$\alpha$$ (heat generation) consequence on the $$\theta \left( \xi \right)$$ temperature profile using $${\text{TiO}}_{2}$$ nanofluid and $${\text{Al}}_{2} {\text{O}}_{3} + {\text{TiO}}_{2}$$ hybrid nanofluid. As the values of the heat generation parameter are enhanced, the fluid temperature goes up considerably. The reason for this is that, the outer heating element transfers additional heat into the nano and hybrid nanofluid flow area, that leads the fluid temperature to rise. The $$\theta \left( \xi \right)$$ temperature profile of the $${\text{TiO}}_{2}$$ nanofluid and $${\text{Al}}_{2} {\text{O}}_{3} + {\text{TiO}}_{2}$$ hybrid nanofluid is elevated owing to the influence of the $$R$$ thermal radiation parameter, as seen in Fig. 12. Enhancement in radiative heat flow promotes the molecular transit inside the framework, and so regular collision between molecules translates into thermal energy. As a result of the increased values of the $$R$$, a greater $$\theta \left( \xi \right)\,$$ temperature distribution has been observed. Furthermore, Fig. 13 exhibits the $$\Phi \left( \xi \right)$$ nanoparticle concentration distribution for varying $$N_{b}$$ values. The increment in factor $$N_{b}$$ corresponds to the possibility of repetitive interactions among $${\text{Al}}_{2} {\text{O}}_{3} ,\,{\text{TiO}}_{2}$$ nanoparticles. As a result, the gap among the nanoparticles shrinks, producing in a reduced $$\Phi \left( \xi \right)$$ concentration distribution. Figure 14 demonstrates the $$\Phi \left( \xi \right)$$ concentration profile of $${\text{TiO}}_{2}$$ nanofluid and $${\text{Al}}_{2} {\text{O}}_{3} + {\text{TiO}}_{2}$$ hybrid nanofluid for varied $$N_{t}$$ values. It has been determined that throughout the scenario of concentration distribution within the boundary layer zone, $$N_{t}$$ operates as a supporting factor. These finding arises owing to the development in thermophoretic processes from a physical point of view. The greater Schmidt number Sc is accountable for reducing the $$\Phi \left( \xi \right)$$ concentration inside the boundary, leading in decreasing the width of the nanoparticle concentration boundary, as shown in Fig. 15. The higher the Sc, the lower the mass diffusivity, and the lower the $$\Phi \left( \xi \right)$$ nanoparticle concentration in the boundary.

### Table discussion

Table 1 visualized the various thermophysical properties of base, nano and hybrid nanofluids. Tables 2, 3 and 4 reveal the impact of varying values of factors involved on the numeric values of various physical quantities including $C_{f} ,C_{g}$, $Nu$, and $Sh$ for engineering purposes. Table 2 shows that $C_{f} ,C_{g}$ is increased when the values of $F_{r} ,k_{1}$ are increased. The $C_{f}$ is decreased, when the values of ${\text{Re}}_{r}$ is increased. Table 3 shows that $Nu$ is increased when the values of $R,\alpha ,\phi_{1} ,\phi_{2}$ are increased. The $Nu$ is decreased, when the values of $\Pr ,S_{t}$ is increased. Table 4 shows that $Sh$ is increased when the values when the values of $Sc,N_{b}$ are increased. The $Sh$ is decreased, when the values of $N_{t}$ is increased. The comparison of the present work with published work^38^ has been shown in Table 5. The current results are found to be in good agreement with those in Ref.^38^ (Supplementary Information).

**Table 2:** Different physical variables have an impact on skin friction, $\left[ {Re_{r} } \right]^{\frac{1}{2}} C_{f} = \frac{1}{{\left( {1 - \phi_{1} - \phi_{2} } \right)^{2.5} }}f^{\prime\prime}\left( 0 \right),\left[ {Re_{r} } \right]^{\frac{1}{2}} C_{g} = \frac{1}{{\left( {1 - \phi_{1} - \phi_{2} } \right)^{2.5} }}g^{\prime}\left( 0 \right)$.

$F_{r}$	$k_{1}$	${\text{Re}}_{r}$	$\frac{1}{{\left( {1 - \phi_{1} - \phi_{2} } \right)^{2.5} }}f^{\prime\prime}\left( 0 \right)$	$\frac{1}{{\left( {1 - \phi_{1} - \phi_{2} } \right)^{2.5} }}g^{\prime}\left( 0 \right)$
0.1	0.5	1.0	$0.3347805$	$1.5327381$
0.3			$0.4537309$	$1.6949731$
0.5			$0.6768929$	$1.7845873$
	0.5		$0.3347805$	$1.5327381$
	0.7		$0.3816925$	$1.6839842$
	0.9		$0.4369237$	$1.7458903$
		1.0	$1.6383981$	$1.5327381$
		1.3	$1.4861693$	$0.5963703$
		1.6	$1.3743643$	$0.6346134$

**Table 3:** Different physical variables have an impact on the Nusselt number $\frac{1}{2}\left[ {Re_{r} } \right]^{\frac{1}{2}} Nu_{x} = - \left( {\frac{{k_{hnf} }}{{k_{f} }} + \frac{4}{3}R\left( {\frac{1}{{\Theta_{w} + S_{t} }} + 1} \right)^{3} } \right)\Theta^{\prime}\left( 0 \right)$.

$\alpha$	$\Pr$	R	$S_{t}$	$\phi_{1} ,\phi_{2}$	$- \left( {\frac{{k_{hnf} }}{{k_{f} }} + \frac{4}{3}R\left( {\frac{1}{{\Theta_{w} + S_{t} }} + 1} \right)^{3} } \right)\Theta^{\prime}\left( 0 \right)$
0.5	7.0	0.2	0.2	0.01	$0.238416$
1.0					$0.425628$
1.5					$0.523737$
	7.0				$0.238416$
	7.5				$0.198695$
	8.0				$0.093632$
		0.2			$0.238416$
		0.4			$0.423835$
		0.8			$0.648986$
			0.2		$0.238416$
			0.5		$0.201476$
			1.0		$0.183431$
				0.01	$0.238416$
				0.02	$0.324386$
				0.03	$0.411374$

**Table 4:** Different physical elements have an impact on the Sherwood number, $\frac{1}{2}\left[ {Re_{r} } \right]^{\frac{1}{2}} Sh = - \Phi^{\prime}\left( 0 \right).$

$N_{t}$	$N_{b}$	$Sc$	$- \Phi^{\prime}\left( 0 \right)$
0.2	0.3	1.0	$1.4352426$
0.4			$1.5368761$
0.6			$1.7917425$
	0.3		$0.3718931$
	0.7		$0.5817817$
	0.9		$0.7690573$
		1.0	$1.1523497$
		1.3	$0.7348537$
		1.5	$0.4835475$

**Table 5:** Comparison of the present work with^38^.

$Fr$	$R$	$f^{\prime\prime}\left( 0 \right)$^38^	$f^{\prime\prime}\left( 0 \right)$ Present	$\Theta^{\prime}\left( 0 \right)$^38^	$\Theta^{\prime}\left( 0 \right)$ Present
0.5	0.2	$0.241035$	$0.241044$	$0.1968238$	$0.1968238$
1.0		$0.346562$	$0.346589$	$0.1968238$	$0.1968238$
1.5		$0.4623723$	$0.4623745$	$0.1968238$	$0.1968238$
	0.2	$0.241035$	$0.241035$	$0.1968238$	$0.1968238$
	0.4	$0.241035$	$0.241035$	$0.24012196$	$0.24012231$
	0.6	$0.241035$	$0.241035$	$0.3365421$	$0.33656421$

Considering common parameters $\alpha = 0.1,\phi_{1} = \phi_{2} = 0,\Pr = 6.2.$

## Conclusions

This work investigates numerically the solution of Darcy–Forchheimer flow of hybrid nanofluid by employing the slip condition. The flow is produced by a swirling the porous disk and is exposed to thermal stratification and nonlinear thermal radiation for controlling the heat transfer of the flow system. The main findings of this research work are:

- There is a decreasing behavior in $f^{\prime}\left( \xi \right),g\left( \xi \right)$ velocity profiles for $\phi_{1} ,\phi_{2} ,\,Fr$ and $k_{1}$.
- Increase in $\phi_{1} ,\phi_{2} ,\,\alpha ,\,R,\,N_{b} \,$ and $N_{t}$ increases the temperature profile.
- The concentration distribution declines for big estimations $Sc$ and Brownian force factor whereas it upsurges for big estimations of thermophoresis parameter.
- The magnitude of the skin friction coefficient increases when the parameters $F_{r} ,k_{1}$ are raised, whereas the parameters ${\text{Re}}_{r}$ have a diminishing impact.
- The magnitude of the rate of heat transfer increases with the factors $R,\alpha$ while it reduces with the factors $\Pr ,N_{b} ,N_{t}$.
- The parameters $Sc,N_{b}$ have a potential to improve the rate of mass transfer in magnitude, meanwhile the factors $Nt$ have the inverse result.
- In future works we include the inclined MHD, Hall effect and activation energy.

## Supplementary Information

Supplementary Information.

## Data Availability

The data that support the findings of this study are available from the corresponding author upon reasonable request.
