# Supplementary material for: Hybrid nanofluid flow through a spinning Darcy–Forchheimer porous space with thermal radiation
Source: Sci Rep. 2021 Aug 18;11:16708. doi: 10.1038/s41598-021-95989-2 (PMC8373955; doi:10.1038/s41598-021-95989-2)
Supplement: Supplementary file 1 — Supplementary Information. [file 41598_2021_95989_MOESM1_ESM.docx]

**Hybrid Nanofluid flow through a Spinning Darcy-Forchheimer Porous Space with Thermal Radiation**

**Nomenclature:**

| Symbol | Discription | Symbol | Discription |
| --- | --- | --- | --- |
| $\left(u,v,w\right)$ | velocity components $\left(m/s\right)$ | $\Omega$ | constant angular velocity |
| $M$ | Magnetic field parameter | $T_{w}$ | Surface temperature $\left(K\right)$ |
| $\rho_{hnf}$ | Density of hybrid nanofluid | $C_{b}$ | drag coefficient |
| $\left(\rho c_{p}\right)_{hnf}$ | volumetric heat capacity of hybrid nanofluid | $d$ | variable permeability |
|  | nanoparticle volume fraction | $d^{*}$ | variable porosity |
| $\theta$ | $\text{Dimensionless temperature }\left(-\right)$ | $T_{0}$ | Reference temperature $\left(K\right)$ |
| $k_{f}$ | thermal conductivity | $\sigma^{*}$ | Stefan Boltzmann constant |
| $G_{r}$ | Grashof number | $K_{\infty}$ | dimensional permeability |
|  | Rosseland approximation | $\varepsilon_{\infty}$ | dimensional porosity |
| $\Pr$ | Prandtl number | $k$ | mean absorption coefficient |
| $C_{f},C_{g}$ | Skin friction coefficient | $T_{\infty}$ | Ambient temperature **$\left(K\right)$** |
| $C_{w}$ | Surface concentration | $\mu_{hnf}$ | dynamic viscosity of hybrid nanofluid |
| $Nu$ | Nusselt number | $g$ | acceleration due to gravity $\left(m/s^{2}\right)$ |
| $\lambda$ | mixed convection or buoyancy parameter | $Q$ | volumetric rate of heat generation/absorption |
| $\rho_{f}$ | Density  | $\nu_{f}$ | Kinematic viscosity  |
| $f'$ | $\text{Dimensionless velocity }\left(-\right)$ | **$\mu_{f}$** | Dynamic viscosity  |
| ,  | volume fraction of **fi**rst and second nanoparticles | $w_{1}$, $w_{2}$ | the first nanoparticle, the second nanoparticle |
| $C_{0}$ | Reference temperature | $Re_{r}$ | Reynolds number |
| *$\left(\rho c\right)_{p}$* | Effective heat capacity of nanoparticles | $\xi$ | Similarity variable |
| $k_{1}$ | Non-dimensional porosity parameter | $T$ | Temperature of the fluid $\left(K\right)$ |
| $\alpha$ | heat source parameter | $\gamma^{*}$ | velocity slip parameter |
| $S_{t}$ | thermal stratification parameter | $\left(\theta_{w}\right)$ | Temperature ratio parameter |
| $Sh$ | Sherwood number | $Sc$ | Schmidt number |
| $k_{hnf}$ | thermal conductivity of hybrid nanofluid | *$C$* | Concentration of the fluid |
| $\Phi$ | $\text{Dimensionless concentration }$ | $C_{\infty}$ | Ambient Concentration |
